# A replication-competent smallpox vaccine LC16m8Δ-based COVID-19 vaccine

**DOI:** 10.1080/22221751.2022.2122580

**Published:** 2022-09-29

**Authors:** Akihiko Sakamoto, Hiroaki Osawa, Hinata Hashimoto, Tetsushi Mizuno, Ammar A. Hasyim, Yu-ichi Abe, Yuto Okahashi, Ryohei Ogawa, Mitsuhiro Iyori, Hisatoshi Shida, Shigeto Yoshida

**Affiliations:** a Laboratory of Vaccinology and Applied Immunology, Kanazawa University School of Pharmacy, Ishikawa, Japan; b Department of Global Infectious Diseases, Graduate School of Medical Sciences, Kanazawa University, Ishikawa, Japan; c Department of Radiology, Faculty of Medicine, Academic Assembly, University of Toyama, Toyama, Japan; d Division of Molecular Virology, Institute of Immunological Science, Hokkaido University, Sapporo, Japan

**Keywords:** BALB/c mouse, cellular immunity, COVID-19, Delta/B.1.617.2 variant of concern, humoral immunity, LC16m8, prime-boost immunization, SARS-CoV-2, emerging and re-Emerging coronaviruses

## Abstract

Viral vectors are a potent vaccine platform for inducing humoral and T-cell immune responses. Among the various viral vectors, replication-competent ones are less commonly used for coronavirus disease 2019 (COVID-19) vaccine development compared with replication-deficient ones. Here, we show the availability of a smallpox vaccine LC16m8Δ (m8Δ) as a replication-competent viral vector for a COVID-19 vaccine. M8Δ is a genetically stable variant of the licensed and highly effective Japanese smallpox vaccine LC16m8. Here, we generated two m8Δ recombinants: one harbouring a gene cassette encoding the severe acute respiratory syndrome coronavirus 2 (SARS-CoV-2) spike (S) glycoprotein, named m8Δ-SARS2(P7.5-S)-HA; and one encoding the S protein with a highly polybasic motif at the S1/S2 cleavage site, named m8Δ-SARS2(P7.5-S_HN_)-HA. M8Δ-SARS2(P7.5-S)-HA induced S-specific antibodies in mice that persisted for at least six weeks after a homologous boost immunization. All eight analysed serum samples displayed neutralizing activity against an S-pseudotyped virus at a level similar to that of serum samples from patients with COVID-19, and more than half (5/8) also had neutralizing activity against the Delta/B.1.617.2 variant of concern. Importantly, most serum samples also neutralized the infectious SARS-CoV-2 Wuhan and Delta/B.1.617.2 strains. In contrast, immunization with m8Δ-SARS2(P7.5-S_HN_)-HA elicited significantly lower antibody titres, and the induced antibodies had less neutralizing activity. Regarding T-cell immunity, both m8Δ recombinants elicited S-specific multifunctional CD8^+^ and CD4^+^ T-cell responses even after just a primary immunization. Thus, m8Δ provides an alternative method for developing a novel COVID-19 vaccine.

## Introduction

Since the outbreak of coronavirus disease 2019 (COVID-19) began, various types of vaccines against this disease, caused by severe acute respiratory syndrome coronavirus 2 (SARS-CoV-2), have been developed worldwide [1–4]. Some of these vaccines have been licensed for clinical use. Although there remain some vaccine-related issues, including immunity waning [5,6] and the appearance of several variants of concern [7–9], vaccines have contributed to the control of the ongoing COVID-19 pandemic.

Viral vectors are a potent vaccine platform for inducing humoral and T-cell immune responses. Among the various viral vectors, replication-deficient ones have been commonly used for the development of COVID-19 vaccines [1–4]. For example, adenoviruses are used as vectors in several COVID-19 vaccines, including AZD1222 (AstraZeneca/University of Oxford), Ad26.COV2.S (Janssen/Johnson & Johnson), and Sputnik V (Gamaleya), all of which have exhibited a high efficacy in clinical trials. The suitability of the modified vaccinia Ankara (MVA) has also been demonstrated in various animal models, including rodents [10–18] and non-human primates [13,14,19]. However, it should be noted that replication-deficient viral vectors can exhibit low immunogenicity in some situations [20].

LC16m8 (m8) is a highly attenuated replication-competent vaccinia virus strain that has a frameshift mutation in the B5R gene [21–23]. Although m8 itself was originally developed and licensed as a smallpox vaccine in Japan, we and other groups have demonstrated its availability as an expression vector for large DNA fragments [22,24–27]. Importantly, a great effort has been made by the Japanese Ministry of Health for the development of m8 to minimize the pathogenicity of the classical replication-competent virus strains [21,22]. Although clinical trials [28] and post-marketing surveillance [29] revealed it to have a high safety profile, it has the drawback of being able to spontaneously generate a more virulent revertant [20]. Therefore, we developed an improved version of m8, called m8Δ, which is a genetically stable variant that lacks the entire B5R gene and can be used as a highly immunogenic vector [20]. Notably, vaccination with a B5R-deficient vaccinia virus [30] was shown to be protective against monkeypox in the recent outbreak [31,32]. Therefore, m8Δ may be a promising platform for a COVID-19 vaccine.

Spike (S) is a glycoprotein responsible for SARS-CoV-2 host cell entry [3,33]. It is expressed on the viral surface and is composed of the S1 and S2 subunits. SARS-CoV-2 entry is initiated by the binding of S1 to the host angiotensin-converting enzyme 2 (ACE2) through its receptor-binding domain (RBD), followed by membrane fusion through the S2 subunit. Therefore, the S protein is used as a main target of COVID-19 vaccines.

The polybasic sequence at the S1/S2 junction is cleaved inside SARS-CoV-2-infected cells by the furin proprotein convertase [3,34,35]. Cleavage of the entry glycoprotein has also been reported in other viruses, including human immunodeficiency virus 1, Ebola virus, and avian influenza viruses [36]. Like in avian influenza viruses [37,38], the cleavability of the S protein contributes to the pathogenicity of SARS-CoV-2 [39]. However, the impacts of the S protein cleavability on the immunogenicity of a COVID-19 vaccine should be further studied.

Here, we show the immunogenicity of our novel m8Δ-based COVID-19 vaccine in mice. The m8Δ variant was engineered to express the SARS-CoV-2 S with or without a highly polybasic motif at the S1/S2 cleavage site. Humoral and cellular immune responses were compared among these vaccine candidates.

## Materials and methods

### Plasmids

The pcDNA3.1 plasmid encoding the Wuhan-Hu-1 SARS-CoV-2 S [40] was obtained from Addgene (145032; Watertown, MA, USA). The typical mutations in the Delta/B.1.617.2 SARS-CoV-2 S [8,9], including T19R, G142D, del157/158, L452R, T478K, D614G, P681R, and D950N, were introduced using the Q5 site-directed mutagenesis kit (New England Biolabs, Ipswich, MA, USA). The pRP plasmid encoding the human ACE2 (RefSeq no. NM_021804.3) under the control of an EF1α promoter and that encoding the human transmembrane protease, serine 2 (TMPRSS2) (RefSeq no. NM_001135099.1) under the control of a CBh promoter were constructed by VectorBuilder (Chicago, IL, USA). For construction of the vaccines, synthetic fragments (GenScript, Piscataway, NJ, USA) encoding the signal peptide of the mouse immunoglobulin (Ig) light chain κ [41], the FLAG tag, the Wuhan-Hu-1 SARS-CoV-2 S (residues 14–1214), and the vesicular stomatitis virus (VSV) G protein-derived membrane anchor [42] were sequentially ligated and inserted into the pVR1 plasmid [43]. For replacement of the S1/S2 cleavage site, the upstream and downstream regions were individually amplified with primers carrying the sequence of *hemagglutinin* (*HA*) from the avian influenza A H5N1 virus. These regions were replaced with the corresponding amplicons, using restriction endonucleases. As a result, the S1/S2 cleavage site was replaced with that of the HA from the H5N1 avian influenza virus (residues 341–351).

### Cells and viruses

Human embryonic kidney (HEK) 293T cells, baby hamster kidney (BHK) cells, and VeroE6 cells expressing human TMPRSS2 [44] were cultured in Dulbecco’s modified Eagle’s medium supplemented with 10% heat-inactivated foetal bovine serum (FBS), non-essential amino acids, penicillin, and streptomycin. Rabbit kidney RK13 cells were cultured in RPMI 1640 medium supplemented with 10% heat-inactivated FBS, penicillin, and streptomycin. HEK293T cells were co-transduced with the pRP plasmid encoding human ACE2 and that encoding the human TMPRSS2, using effectene (Qiagen, Hilden, Germany). Stable transfectants were isolated by using the selection markers.

Canarypox virus and m8Δ were previously described [20]. As a backbone of the pseudovirus, the G protein-pseudotyped recombinant VSV carrying the luciferase gene [45] was purchased from Kerafast (Boston, MA, USA). The SARS-CoV-2 Wuhan strain JPN/TY/WK-521 [46,47] and the Delta/B.1.617.2 strain TY11-927 [47] were provided by the National Institute of Infectious Diseases in Japan. SARS-CoV-2 was propagated and titrated as previously described [8].

### Construction of the m8Δ-based vaccines

The m8Δ-based vaccines were constructed as previously described, with some modifications [26]. BHK cells were first inoculated with canarypox virus at a multiplicity of infection (MOI) of 10 and then transduced with a mixture of DNA from m8Δ [20] and the pVR1 encoding the vaccine construct, using lipofectamine LTX (Thermo Fisher Scientific, Waltham, MA, USA). The resulting recombinant virus was released from the cells through five freeze–thaw cycles. The m8Δ encoding the vaccine construct was isolated through the repetitive inoculation of RK13 cells with the crude virus, followed by plaque purification. The loss of HA expression was confirmed by using peripheral blood from a white leghorn (Shimizu Laboratory Supplies, Kyoto, Japan), as previously described [43]. The isolated clone was propagated in RK13 cells at 33°C and purified through a 20% – 40% sucrose gradient. The successful insertion of the vaccine construct was confirmed by PCR, conducted using the following primer pairs: 5′-ATGACACGAT TACCAATACT TTTG-3′ (F1) and 5′-AGTCTCGTCT GTTGTGGATT CTCC-3′ (R1); and 5′-GGGGTTGCGA TGTTAAACGG-3′ (F2) and 5′-GGCTGGGACA CGTACTCAAA-3′ (R2).

### Mice

All animal experiments were performed using 7- to 8-week-old female mice with the approval of the Animal Care and Use Committee of Kanazawa University (AP-214212). BALB/c mice were purchased from SLC (Shizuoka, Japan). Immunization with the recombinant vaccinia virus was performed, as previously described [27,48–50]. Briefly, a 10-µL drop containing 1×10^7^ plaque-forming units of virus in 5% D-sorbitol and 5% peptone was placed on the inoculation skin site, which was then scarified with a bifurcated needle, endeavouring to stay within the superficial epidermis. All efforts were made to minimize animal suffering during the experiments.

## Results

### Construction of the m8Δ-based vaccines

**Figure 1.**
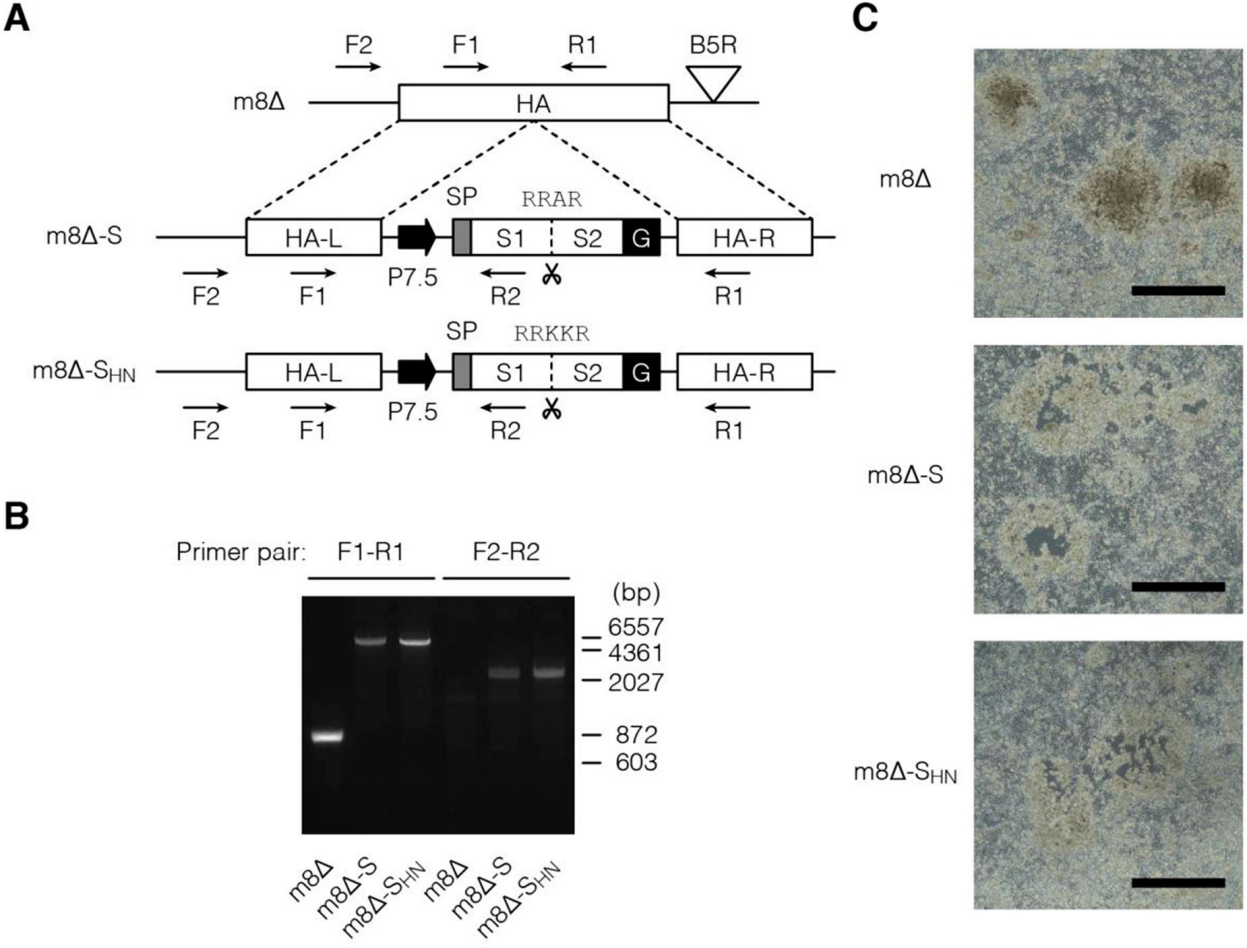
Construction of the m8Δ-based vaccines. (A) Schematic of the vaccines. Characteristic polybasic sequences are listed above the S1/S2 cleavage sites, indicated by scissors. Arrows indicate the primers used for validation by PCR. m8Δ-S, m8Δ-SARS2(P7.5-S)-HA; m8Δ-S_HN_, m8Δ-SARS2(P7.5-S_HN_)-HA; SP, signal peptide; G, VSV G protein-derived membrane anchor; L, left arm; R, right arm. (B) Validation of the recombinant viruses by conducting PCR with the indicated primer pairs. The indicated viral DNA was used as the template. bp, base pair. (C) Loss of HA expression in the recombinant viruses. Three days after RK13 cells were inoculated with the indicated recombinant viruses at a dose of 50–100 plaque-forming units per 7.5×10^5^ cells, they were used in a haemadsorption assay. Data are representative from two independent experiments with similar results. Scale bars, 1 mm.

Here, m8Δ [20] was engineered to express the ectodomain of the Wuhan-Hu-1 SARS-CoV-2 S tethered by a VSVG-derived membrane anchor [42], which was driven under the control of a 7.5-kD promoter (P7.5) [51] (Figure 1(A)). To explore the impact of the S1/S2 cleavage, the corresponding site was replaced with that of the HA from the H5N1 avian influenza virus [38]. These constructs were inserted into the HA locus [43] of m8Δ by performing homologous recombination. The resulting viruses, named m8Δ-SARS2(P7.5-S)-HA and m8Δ-SARS2(P7.5-S_HN_)-HA, respectively, were plaque-purified and validated by PCR (Figure 1(B)). Because the constructs were inserted into the *HA* locus, the purity of these recombinant viruses was ascertained by using a haemadsorption assay [43] (Figure 1(C)).

### *In vitro* expression of the S protein

**Figure 2.**
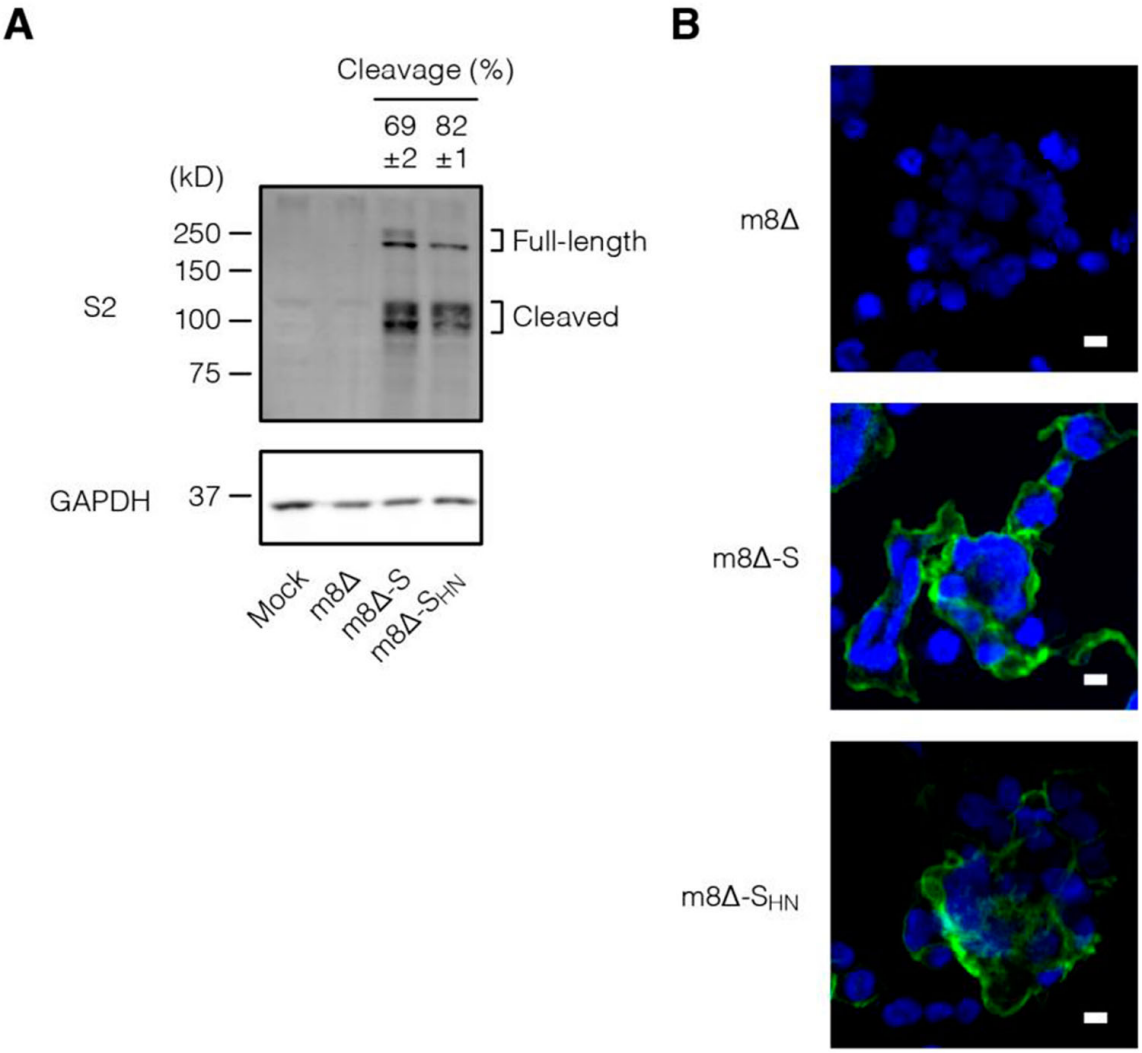
*In vitro* expression of the S protein. (A) Immunoblotting of HEK293T cells that were inoculated with the indicated recombinant viruses at an MOI of 1 or mock infected one day before the analysis. Antibody targets are shown on the left side of the panels. The cleaved form frequency was calculated via densitometry from three independent experiments and is shown as the mean±SEM. Glyceraldehyde 3-phosphate dehydrogenase (GAPDH) was used as a loading control. m8Δ-S, m8Δ-SARS2(P7.5-S)-HA; m8Δ-S_HN_, m8Δ-SARS2(P7.5-S_HN_)-HA. (B) Immunofluorescence assays of HEK293T cells that were inoculated with the indicated viruses at an MOI of 0.1 one day before the analysis. SARS-CoV-2 S2 (green) and nuclei (blue) were visualized on the non-permeabilized formalin-fixed cells. Data are representative of three (A) or at least two (B) independent experiments. Scale bars, 10 µm.

The expression of the S protein was examined *in vitro*. To this end, HEK293T cells were inoculated with the m8Δ-based vaccines. Immunoblotting revealed the expression of the S protein in both its full-length (∼200 kD) and cleaved forms (∼100 kD) [52] (Figure 2(A) and Supplementary Figure 1). According to the densitometry data, the S_HN_ protein was cleaved more efficiently compared with the normal S protein. Furthermore, both types of S protein were expressed on the cell surface, as indicated by the immunofluorescence assay data (Figure 2(B)). These results indicate that the S protein was efficiently synthesized by both m8Δ-SARS2(P7.5-S)-HA and m8Δ-SARS2(P7.5-S_HN_)-HA and then transported to the cell surface.

### Humoral immune responses to the m8Δ-based vaccines

**Figure 3.**
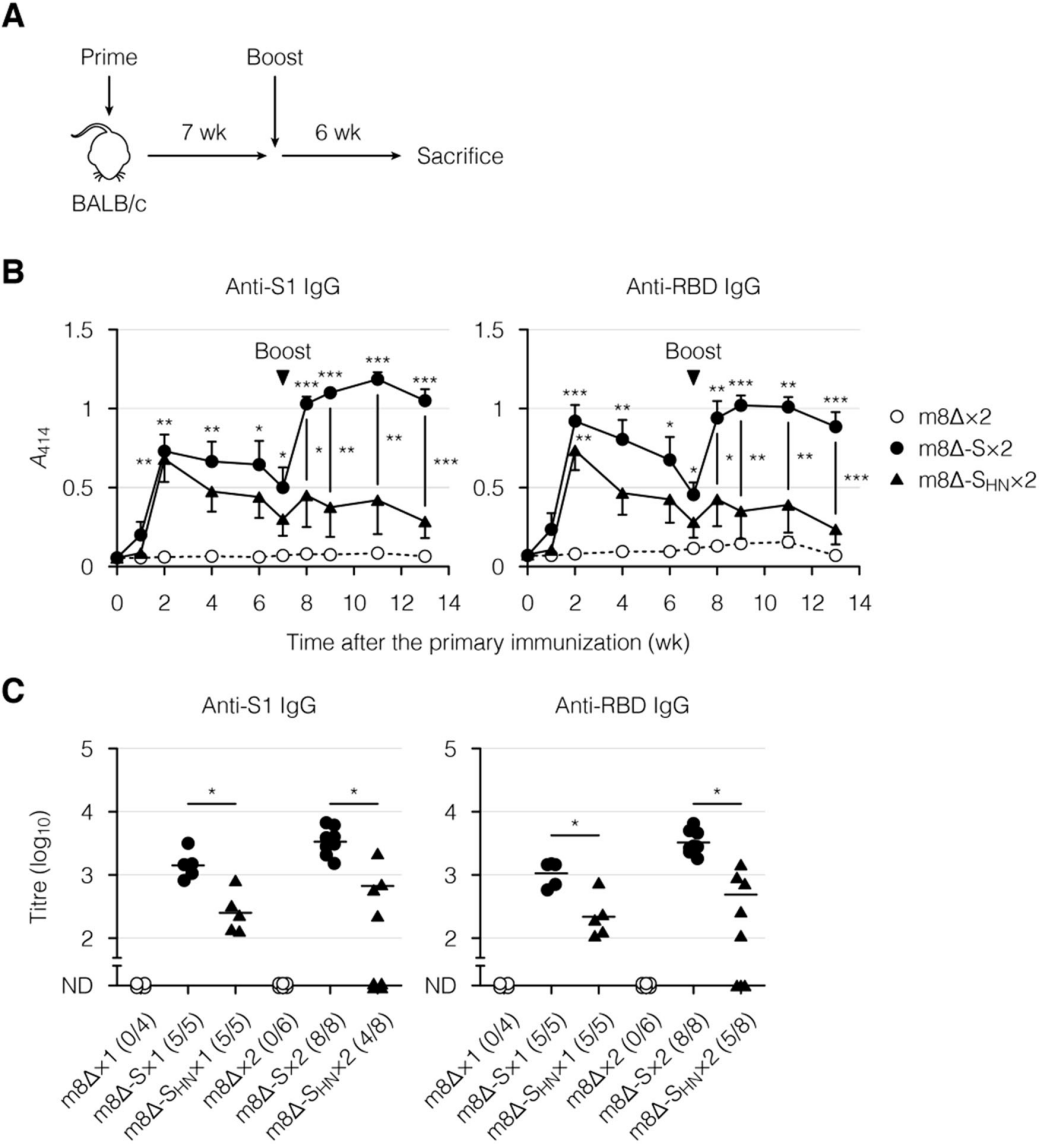
Antibody production induced by immunization with the m8Δ-based vaccines. (A) Experimental outline. (B) Time course of the antibody levels after primary immunization with the indicated virus. The antibody levels were measured by ELISA using diluted serum samples (1:200). The dilution factor of 200 corresponded to the 50% effective concentration of the serum samples obtained six weeks after the boost immunization. Symbols and bars represent the means and SEM, respectively. *A*_414_, absorbance at 414 nm; m8Δ-S, m8Δ-SARS2(P7.5-S)-HA; m8Δ-S_HN_, m8Δ-SARS2(P7.5-S_HN_)-HA. (C) Endpoint titres of the indicated antibodies six weeks after the primary (×1) or boost (×2) immunization. Titres of <100 are listed as not determined (ND). The proportions of samples with measurable titres are shown in parentheses. Each symbol represents an individual mouse. Horizontal lines represent geometric means. Data were pooled from three independent experiments using four or more mice per experimental group. Data at each time point in (B) were analysed by Tukey test. The statistical significance of the difference from the m8Δ×2 group is indicated by * near the individual symbols. The log-transformed data in (C) were analysed by a Welch *t*-test. ***P < 0.001, **P < 0.01, *P < 0.05.

Next, the immunogenicity of the m8Δ-based vaccines was examined in BALB/c mice. These vaccines were injected through superficially injured skin [48–50] two separate times, seven weeks apart (Figure 3(A)). As a control, parental m8Δ was injected similarly. Blood was collected serially for the analysis of humoral immunity. The time course of induced antibody levels was examined by ELISA (Figure 3(B) and Supplementary Figure 2(A)). The antibody levels peaked two weeks after the primary immunization with the m8Δ-based vaccines and then gradually decreased. The antibody levels were increased at one week after the boost immunization with m8Δ-SARS2(P7.5-S)-HA, and these higher levels were sustained for at least five weeks. In contrast, the antibody levels were poorly boosted by the second immunization with m8Δ-SARS2(P7.5-S_HN_)-HA. The antibody levels were also measured by an endpoint titration (Figure 3(C) and Supplementary Figure 2(B)). The S1- and RBD-specific IgG titres were lower in the mice immunized with m8Δ-SARS2(P7.5-S_HN_)-HA. Furthermore, these differences were even larger when the S1- and RBD-specific IgG titres were normalized to the S2-specific IgG titres (Supplementary Figure 2(C,D)). In contrast, boost immunization with m8Δ-SARS2(P7.5-S)-HA resulted in S1- and RBD-specific IgG titres that were 2.4- and 3.1-times higher, respectively (Figure 3(C)). Thus, promising levels of antibodies were elicited by prime-boost immunization with m8Δ-SARS2(P7.5-S)-HA but not with m8Δ-SARS2(P7.5-S_HN_)-HA.

**Figure 4.**
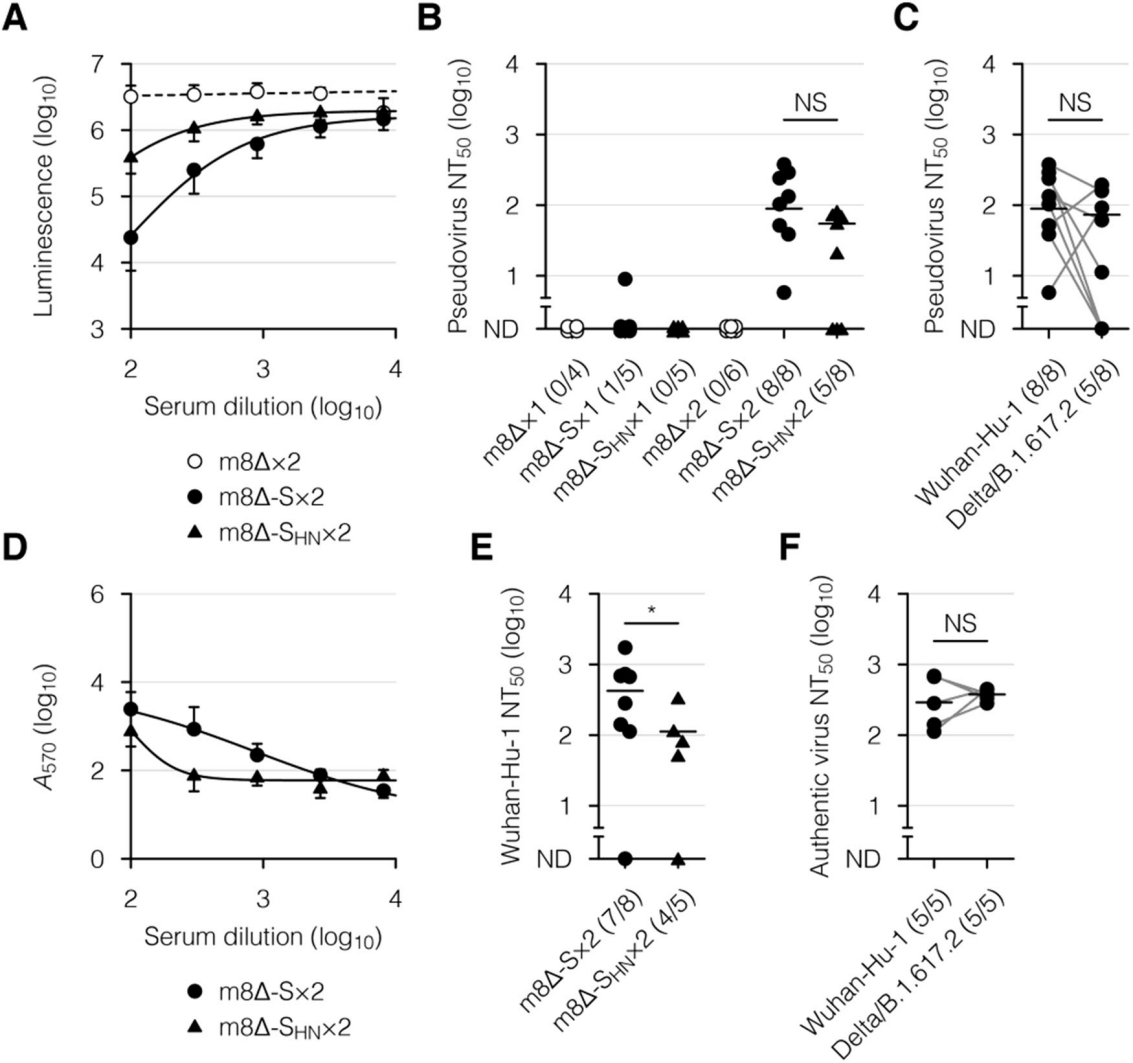
Neutralizing activity of the serum after immunization with the m8Δ-based vaccines. (A) Neutralization of the Wuhan-Hu-1 pseudovirus by the serum samples in a concentration-dependent manner, as determined by luciferase assay. The pseudovirus was incubated with the serum samples obtained six weeks after the boost (×2) immunization with the indicated virus. m8Δ-S, m8Δ-SARS2(P7.5-S)-HA; m8Δ-S_HN_, m8Δ-SARS2(P7.5-S_HN_)-HA. (B) Neutralizing activities of the serum samples obtained six weeks after the primary (×1) or boost immunization with the indicated virus. The proportions of samples with measurable neutralizing activity are shown in parentheses. NT_50_, 50% neutralizing concentration; ND, not determined. (C) Neutralizing activities of the serum samples described in (B) obtained six weeks after the boost immunization with m8Δ-S, as measured using the indicated pseudovirus. (D) Neutralization of the Wuhan SARS-CoV-2 by the serum samples described in (A) in a concentration-dependent manner, as determined by crystal violet assay. Serum samples lacking neutralizing activity against the Wuhan-Hu-1 pseudovirus were excluded. *A*_570_, absorbance at 570 nm. (E) Neutralizing activities of the serum samples described in (D). (F) Neutralizing activities of the serum samples described in (C) against the indicated SARS-CoV-2. Serum samples displaying neutralizing activity against the corresponding pseudovirus were analysed. Symbols and bars in (A,D) represent the means and SEM, respectively. Each symbol in (B,C,E,F) represents an individual mouse or patient. The same serum samples are linked in (C,F). Horizontal lines in (B,C,E,F) represent the geometric means. Data were pooled from three independent experiments using four or more mice per experimental group. The log-transformed data in (B,C,E,F) were analysed by an unpaired (B,E) or paired Welch *t*-test (C,F). *P < 0.05. NS, not statistically significant.

The neutralizing activity of the serum was then examined, using a VSV-based pseudovirus [53,54] and infectious SARS-CoV-2. The pseudovirus was engineered to express the Wuhan-Hu-1 or Delta/B.1.617.2 SARS-CoV-2 S [8,9] on the viral surface and induce the expression of luciferase in infected cells. When the Wuhan-Hu-1 SARS-CoV-2 S-pseudotyped virus was incubated with the serum samples from mice immunized with m8Δ-SARS2(P7.5-S)-HA, the luciferase expression was suppressed in a concentration-dependent manner (Figure 4(A)). The pseudovirus was also validated, using serum samples from COVID-19 patients (Supplementary Figure 3). Sufficient levels of neutralizing activity were identified after the second immunization with m8Δ-SARS2(P7.5-S)-HA but not m8Δ-SARS2(P7.5-S_HN_)-HA (Figure 4(B)). More than half of the samples from mice immunized with m8Δ-SARS2(P7.5-S)-HA displayed neutralizing activity against the Delta/B.1.617.2 variant of concern (Figure 4(C)). When Wuhan SARS-CoV-2 was incubated with these serum samples, the cytopathic effect was suppressed in a concentration-dependent manner (Figure 4(D)). Sufficient levels of neutralizing activity were identified after the second immunization with m8Δ-SARS2(P7.5-S)-HA but not m8Δ-SARS2(P7.5-S_HN_)-HA (Figure 4(E,F)). Thus, a promising level of humoral immunity was elicited by the prime-boost immunization with m8Δ-SARS2(P7.5-S)-HA.

### Cellular immune responses to the m8Δ-based vaccines

**Figure 5.**
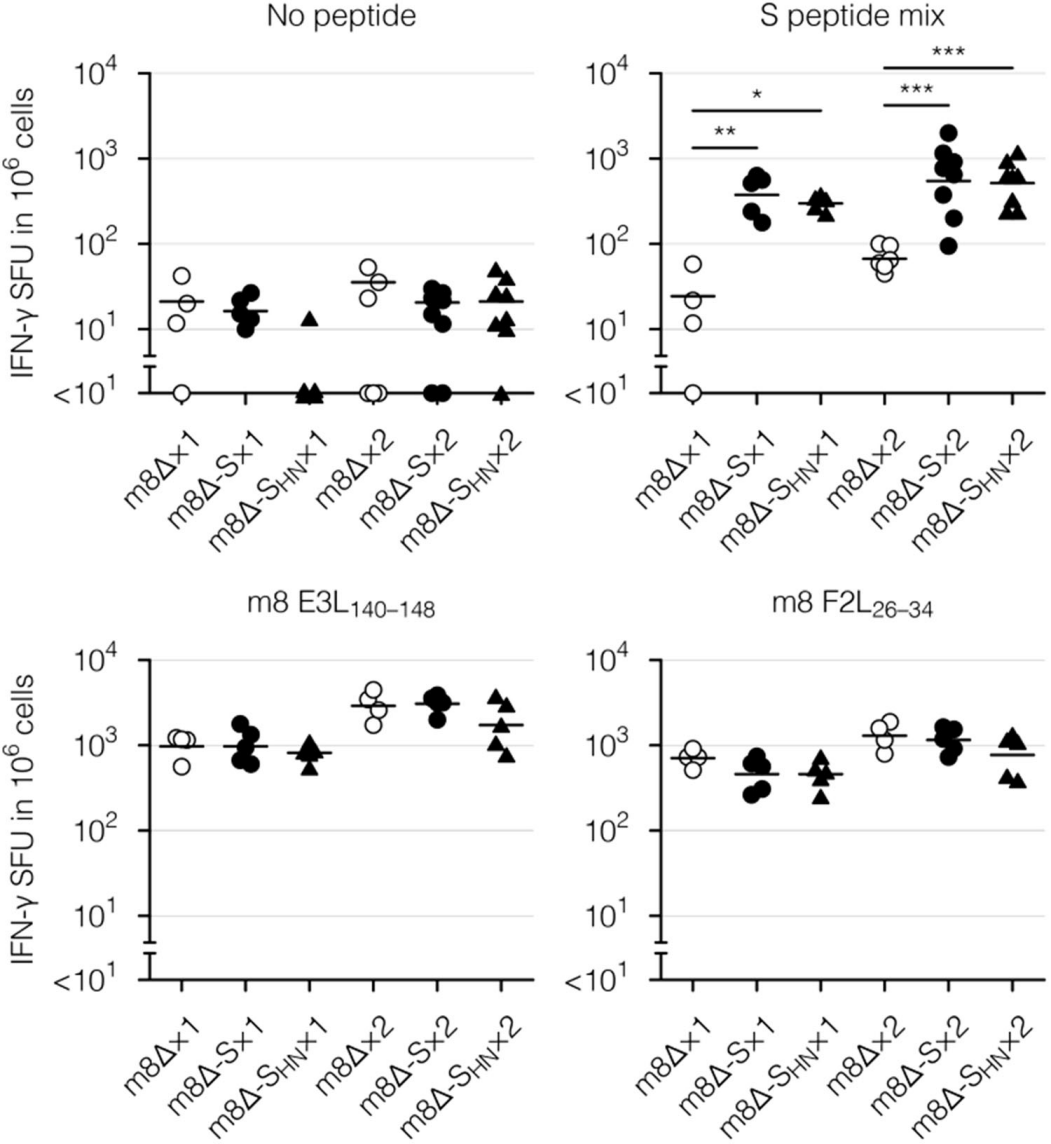
Cellular immune responses to the m8Δ-based vaccines as measured by an ELISpot assay. Splenocytes were obtained six weeks after the primary (×1) or boost (×2) immunization with the indicated virus. IFN-γ spot-forming units (SFU) were measured after *in vitro* stimulation with or without the indicated peptide. Each symbol represents an individual mouse. Horizontal lines represent the geometric means. Data were pooled from two or three independent experiments using four or more mice per experimental group. m8Δ-S, m8Δ-SARS2(P7.5-S)-HA; m8Δ-S_HN_, m8Δ-SARS2(P7.5-S_HN_)-HA. The log-transformed data from after the primary or boost immunization were analysed individually by a Tukey test. Combinations without mark are not statistically different. ***P < 0.001, **P < 0.01, *P < 0.05.

Finally, cellular immunity was examined with an enzyme-linked immunospot (ELISpot) assay and intracellular cytokine staining. To this end, splenocytes were stimulated with a mixture of peptides spanning the entirety of the S protein [17,55] or with the S_268–276_ peptide as a representative CD8^+^ T-cell epitope [12,56]. Vaccinia virus-derived E3L_140–148_ and F2L_26–34_ peptides [57] were used as a control for parental m8Δ and the m8Δ-based vaccines. The peptide-reactive cells were identified as interferon (IFN)-γ-secreting cells in an ELISpot assay. Vaccinia virus-specific cells were identified after immunization with parental m8Δ or the m8Δ-based vaccines (Figure 5), suggesting that the recombinant viruses were successfully injected. In contrast, S protein-specific cells were identified only after immunization with the m8Δ-based vaccines (Figure 5 and Supplementary Figure 4(A)). Boost immunization with m8Δ-SARS2(P7.5-S)-HA or m8Δ-SARS2(P7.5-S_HN_)-HA resulted in the formation of 1.4- or 1.7-times more S protein-specific cells, respectively, compared with the pre-boost immunization level. Thus, the levels of elicited cellular immunity were similar following immunization with both types of m8Δ-based vaccines.

**Figure 6.**
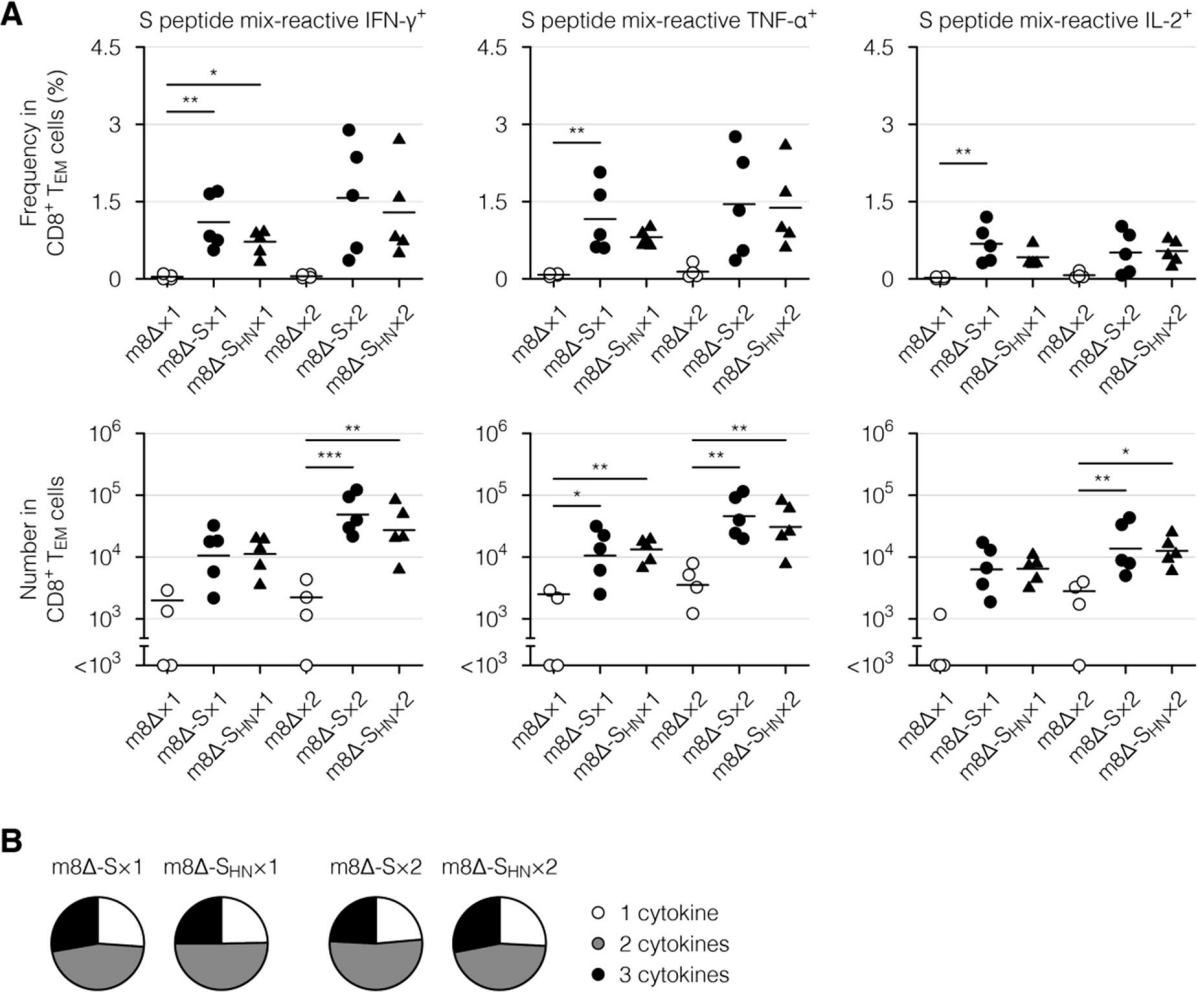
Cytokine profiles of the CD8^+^ memory T cells. (A) Frequencies and absolute numbers of the CD8^+^ effector memory T (T_EM_) cells reactive to the S peptide mix, as measured by intracellular cytokine staining. Splenocytes were obtained six weeks after the primary (×1) or boost (×2) immunization with the indicated virus. The gating strategy applied to isolate the CD8^+^ T_EM_ cells is shown in Supplementary Figure 5. Cytokine-producing cells were identified as shown in Supplementary Figure 6(A). Each symbol represents an individual mouse. Horizontal lines represent the means (upper panels) or geometric means (lower panels). Data were pooled from two independent experiments using four or more mice per experimental group. m8Δ-S, m8Δ-SARS2(P7.5-S)-HA; m8Δ-S_HN_, m8Δ-SARS2(P7.5-S_HN_)-HA. (B) Percentages of the S peptide mix-reactive CD8^+^ T_EM_ cells described in (A) that expressed the indicated number of cytokines among IFN-γ, TNF-α, and IL-2. The data shown in (A, upper panels) and the log-transformed data shown in (A, lower panels) after the primary and boost immunization were analysed individually by a Tukey test. Combinations without mark are not statistically different. ***P < 0.001, **P < 0.01, *P < 0.05.

**Figure 7.**
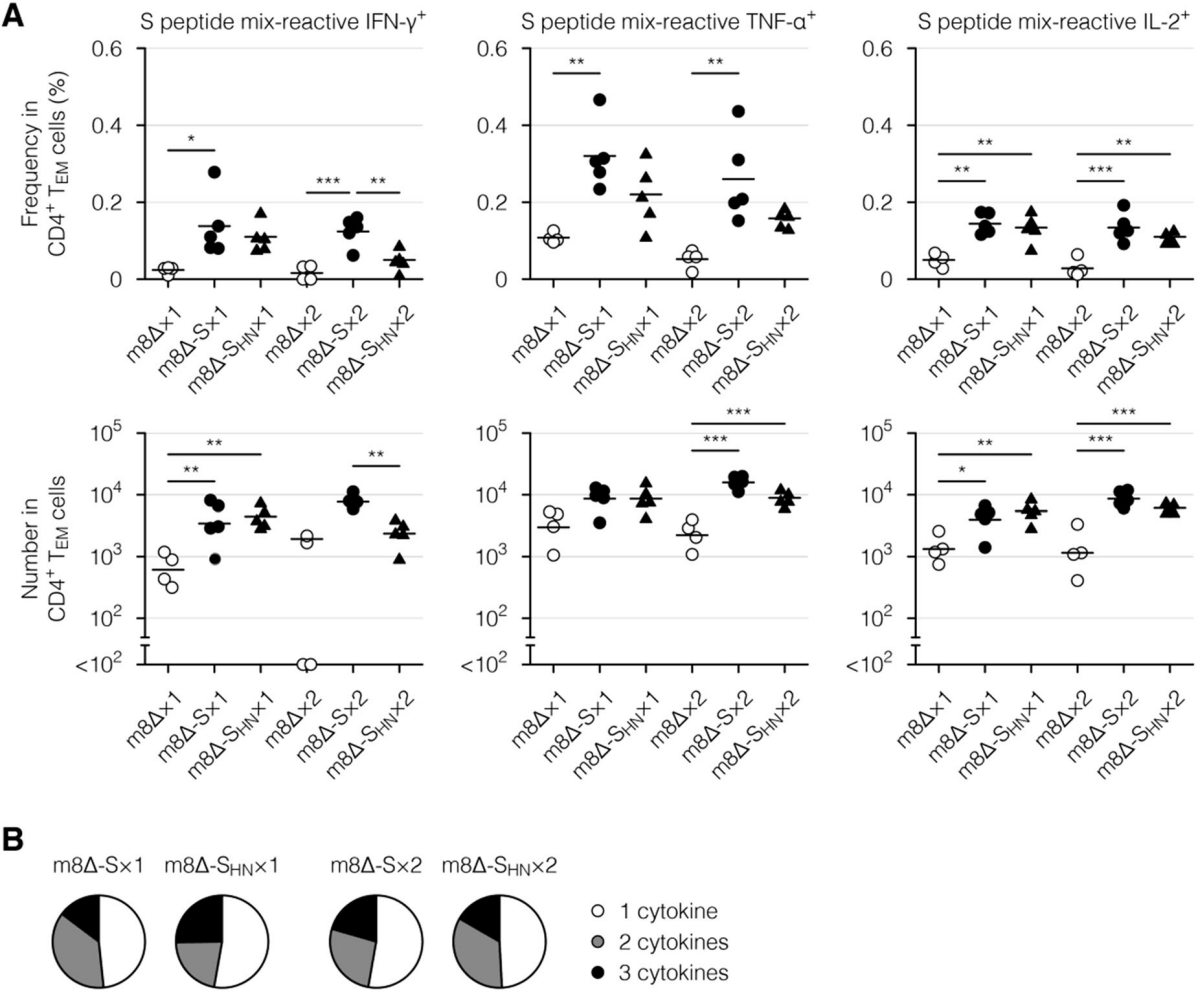
Cytokine profiles of the CD4^+^ memory T cells. (A) Frequencies and absolute numbers of the CD4^+^ effector memory T (T_EM_) cells reactive to the S peptide mix, as measured by intracellular cytokine staining. Splenocytes were obtained six weeks after the primary (×1) or boost (×2) immunization with the indicated virus. The gating strategy applied to isolate the CD4^+^ T_EM_ cells is shown in Supplementary Figure 5. The cytokine-producing cells were identified as shown in Supplementary Figure 6(B). Each symbol represents an individual mouse. Horizontal lines represent the means (upper panels) or geometric means (lower panels). Data were pooled from two independent experiments using four or more mice per experimental group. m8Δ-S, m8Δ-SARS2(P7.5-S)-HA; m8Δ-S_HN_, m8Δ-SARS2(P7.5-S_HN_)-HA. (B) Percentages of the S peptide mix-reactive CD4^+^ T_EM_ cells described in (A) that expressed the indicated number of cytokines among IFN-γ, TNF-α, and IL-2. The data shown in (A, upper panels) and the log-transformed data shown in (A, lower panels) after the primary and boost immunization were analysed individually by a Tukey test. Combinations without mark are not statistically different. ***P < 0.001, **P < 0.01, *P < 0.05.

Next, cytokine profiles of the effector memory T cells, which are the main responding subset [17], were examined via intracellular cytokine staining. Primary immunization with the m8Δ-based vaccines resulted in the formation of CD8^+^ (Figure 6(A), Supplementary Figure 4(B), and Supplementary Figure 7(A)) and CD4^+^ effector memory T cells (Figure 7(A) and Supplementary Figure 7(B)) with the potential to produce cytokines, including IFN-γ, tumour necrosis factor-α (TNF-α), and interleukin-2 (IL-2). Similar functionality was identified in CD8^+^ (Figure 6(B), Supplementary Figure 4(C,D), and Supplementary Figure 8(A)) and CD4^+^ effector memory T cells (Figure 7(B) and Supplementary Figure 8(B)) from mice immunized with either type of m8Δ-based vaccine. These cytokine profiles were only slightly boosted by the second immunization (Figure 6, Figure 7, Supplementary Figure 4(B – D), and Supplementary Figure 8). Thus, primary immunization resulted in the formation of multifunctional effector memory T cells, while the impact of the boost immunization on these cells was milder than that on humoral immunity.

## Discussion

Here, we showed the immunogenicity of our m8Δ-based COVID-19 vaccine in mice. The m8Δ variant encoding the SARS-CoV-2 S protein induced S-specific antibodies that persisted for at least six weeks after a homologous boost immunization. Neutralizing activity against the Wuhan-Hu-1 pseudovirus was detected in all the analysed serum samples, and that against the Delta/B.1.617.2 pseudovirus was still identified in over half of the samples. Importantly, most serum samples also neutralized the infectious SARS-CoV-2 Wuhan and Delta/B.1.617.2 strains. These humoral immune responses were significantly weaker in the mice immunized with the vaccine containing a substitution of the S1/S2 cleavage site. Regardless of this substitution, even primary immunization with these m8Δ-based COVID-19 vaccines induced the formation of CD8^+^ and CD4^+^ effector memory T cells with the functionality to produce multiple cytokines. Thus, m8Δ provides an alternative approach for developing a novel COVID-19 vaccine.

Vaccinia virus is characterized by its high capacity for inserted DNA and its high and durable immunogenicity [21,58,59]. The suitability of vaccinia virus strains, including the replication-competent m8 and m8Δ and the replication-deficient MVA, as a vaccine platform has been extensively studied. Among these strains, m8 is characterized by its high immunogenicity (>500 times more immunogenic than MVA [20]) and safety profile (no severe complications after use in vaccination of >50,000 Japanese children [22,28]). Consistent with those findings, our m8Δ-based vaccine successfully elicited a neutralizing antibody against the SARS-CoV-2 pseudovirus and infectious SARS-CoV-2. Additionally, vaccination with m8Δ [27] should be protective against monkeypox, the recent outbreak of which is concerning [28,29]. Although we observed that the induced humoral and cellular immune responses were sustained for at least six weeks after the second immunization, the durability of these responses for a longer period should be further examined in the future.

The second immunization impacted humoral and cellular immune responses differently. Antibody levels were rapidly boosted by the second immunization, suggesting the contribution of memory B cells formed following the primary immunization. Neutralizing activity was identified only after the second immunization, suggesting that the maturation of B cells is required. Thus, the second immunization was important for the improvement of humoral immunity. In contrast, only mild impacts of the second immunization on the formation and functionality of effector memory T cells were identified. However, because cellular immunity was examined only using a mixture of peptides spanning the whole region of the S protein [17,55], an epitope-level analysis may be necessary for obtaining a full understanding of the cellular immunity impacts.

Humoral, but not cellular, immunity was negatively influenced by the cleavability of the S protein in our m8Δ-based vaccine. When the S1/S2 cleavage site was replaced to increase its cleavability, the antibody levels and neutralizing activity, especially those induced by the second immunization, were lower. In contrast, no significant impact in the formation and functionality of effector memory T cells was identified. One explanation may be that the S1/S2 junction is important for the stability of the S protein [3]. Notably, the S1/S2 junction is mutated in the recent SARS-CoV-2 variants, including the Delta/B.1.617.2 and Omicron/B.1.1.529 variants [7–9]. When m8Δ is engineered to express the S protein of these variants, modification of the S1/S2 junction should be considered.

In conclusion, we showed the suitability of the m8Δ variant as a vector platform for a COVID-19 vaccine. Homologous prime-boost immunization with our vaccine elicited durable neutralizing antibody and achieved the formation of multifunctional effector memory T cells in mice. The immunogenicity of our vaccine will be improved by considering the experimental conditions, including the dose and the interval between prime-boost immunization, or modifying an immunoregulatory gene within the vaccine backbone [58] in the future. Moreover, our vaccine has a great potential to exert all its powers in humans because m8Δ is a human virus that has been widely used in clinical settings for ages.

## Supplementary Material

Supplemental Material

## Data Availability

The authors confirm that the data supporting the findings of this study are available within the article and its supplementary materials.
