# Supplementary material for: A replication-competent smallpox vaccine LC16m8Δ-based COVID-19 vaccine
Source: Emerg Microbes Infect. 2022 Sep 29;11(1):2359–70. doi: 10.1080/22221751.2022.2122580 (PMC9527789; doi:10.1080/22221751.2022.2122580)
Supplement: Supplemental Material [file TEMI_A_2122580_SM4812.docx]

# Supplementary materials and methods

## Immunoblotting

Cells were lysed with Laemmli buffer containing 1% 2-mercaptoethanol, boiled, and subjected to sodium dodecyl sulfate-polyacrylamide gel electrophoresis. N-glycans were removed using peptide-N_4_-(*N*-acetyl-β-D-glucosaminyl)asparagine amidase F (New England Biolabs), in accordance with the manufacturer’s instructions. Immunoblotting was performed as previously described [1], using the following antibodies: anti-SARS-CoV-2 S2 rabbit polyclonal IgG (40590-T62, 1:3000; Sino Biological, Wayne, PA, USA), anti-glyceraldehyde 3-phosphate dehydrogenase mouse IgG1 (clone no. 5A12, 0.1 µg/mL; FUJIFILM Wako Pure Chemical, Osaka, Japan), anti-rabbit IgG IRDye 680LT (926-68021, 0.05 µg/mL; LI-COR, Lincoln, NE, USA), and anti-mouse IgG IRDye 800CW (926-32210, 0.05 µg/mL; LI-COR). Bands were visualized on an Odyssey infrared imager (LI-COR) and analysed with ImageJ software [2].

## Immunofluorescence assay

The immunofluorescence assay was performed on an eight-well chamber slide (Thermo Fisher Scientific) coated with poly-L-lysine (Merck, Darmstadt, Germany). Cells were fixed with 10% neutral-buffered formalin solution (Merck) and serially incubated with anti-SARS-CoV-2 S2 rabbit polyclonal IgG (40590-T62, 1:100; Sino Biological) and anti-rabbit IgG Alexa Fluor 488 (A-11008, 10 µg/mL; Thermo Fisher Scientific). The chamber was mounted with a drop of Vectashield containing 4,6ʹ-diamidino-2-phenylindole (Vector Laboratories, Burlingame, CA, USA). Images were acquired on a BZ-X710 fluorescence microscope (Keyence, Tokyo, Japan).

## Enzyme-linked immunosorbent assay (ELISA)

Serum was separated from mouse blood samples collected from the tail vein or heart. If necessary, anaesthesia was performed via an intramuscular injection with ketamine and xylazine. Serum samples collected from 18 COVID-19 patients (mixed sex population; 40–90 years old; median, 70.5 years old) 33–34 days after their diagnosis via PCR and those from healthy donors (mixed sex population; 18–47 years old; median, 34 years old) were purchased from RayBiotech (Peachtree Corners, GA, USA). No patients were vaccinated before diagnosis. Patient samples were collected in May, 2020, while healthy controls were collected before the COVID-19 outbreak. All serum samples were inactivated with heat or 4% Triton X-100 before use. The following antibodies were used for validation: anti-SARS-CoV-2 S1 RBD human IgG1 (clone no. 414-4; BioLegend, San Diego, CA, USA) [3], anti-SARS-CoV-2 S1 N-terminal domain rabbit polyclonal IgG (ab275759; Abcam, Cambridge, UK), anti-SARS-CoV-2 S2 mouse IgG2b (clone no. A20085C; BioLegend), and anti-SARS-CoV-2 nucleocapsid mouse IgG2b (clone no. A20087F; BioLegend). For ELISAs, 96-well plates were coated with recombinant SARS-CoV-2 S1 (4 µg/mL; Sino Biological), S2 (4 µg/mL; Sino Biological), or RBD (4 µg/mL; GenScript). Serially diluted serum samples were loaded in duplicate, followed by their incubation with anti-mouse (170-6516, 1:2000; Bio-Rad Laboratories, Hercules, CA, USA), anti-human (172-1050, 1:2000; Bio-Rad Laboratories), or anti-rabbit IgG horseradish peroxidase conjugates (170-6515, 1:2000; Bio-Rad Laboratories). Endpoint titres were analysed on a Multiskan FC microplate reader (Thermo Fisher Scientific), as previously described [1].

## Pseudovirus neutralization assay

Pseudovirus was produced and titrated as previously described [4,5]. Briefly, cell culture dishes were coated with Cellmatrix type I-C collagen (Nitta Gelatin, Osaka, Japan). HEK293T cells were transduced with pcDNA3.1 encoding the Wuhan-Hu-1 [6] or Delta/B.1.617.2 SARS-CoV-2 S [7,8], using effectene. One day later, the cells were inoculated for 2 h with the VSV backbone at the 50% tissue culture infectious dose (TCID_50_) of 8×10^4^ per 10^6^ cells and cultured overnight in fresh medium. Culture supernatants were then harvested as the pseudovirus and stored at −80 °C until use. For the neutralization assay, the pseudovirus was incubated with serially diluted serum samples in triplicate for 1 h at 37 °C. Freshly trypsinized HEK293T cells stably co-expressing human ACE2 and human TMPRSS2 were then mixed with the pseudovirus at the TCID_50_ of 3×10^2^ per 2×10^4^ cells and cultured overnight. The luciferase expression was measured on a GloMax 96 microplate luminometer (Promega, Madison, WI, USA) using the luciferase assay system (Promega).

## Virus neutralization assay

The virus neutralization assay was performed in a biosafety level 3 laboratory. SARS-CoV-2 was incubated with serially diluted serum samples in triplicate for 1 h at 37 °C. VeroE6 cells expressing human TMPRSS2 were then inoculated with this mixture at the TCID_50_ of 50 per 2×10^4^ cells and cultured for 3 d. The surviving cells were fixed in paraformaldehyde, stained with 0.1% crystal violet in 10% ethanol, and lysed in 1% sodium dodecyl sulfate. Neutralizing activities were analysed on a Multiskan GO microplate reader (Thermo Fisher Scientific), as previously described [9].

## Enzyme-linked immunospot (ELISpot) assay

ELISpot assays were performed using the mouse interferon (IFN)-γ ELISpot set (BD Biosciences, San Jose, CA, USA). Single-cell suspensions from the spleen were deprived of red blood cells and stimulated overnight with or without either of the following peptides in RPMI 1640 medium supplemented with 10% heat-inactivated FBS, penicillin, and streptomycin: 1 µg/mL LC16m8 E3L_140–148_ (residues 140–148) or F2L_26–34_ in dimethyl sulfoxide (DMSO) as a CD8^+^ T-cell epitope [10]; 1 µg/mL PepMix SARS-CoV-2 S peptide pool 1+2 (JPT Peptide Technologies, Berlin, Germany) in DMSO as a CD8^+^ and CD4^+^ T-cell epitope mixture [11]; or 5 µg/mL SARS-CoV-2 S_268–276_ in DMSO/H_2_O (4:1, v/v) as a CD8^+^ T-cell epitope that we and other groups [12,13] predicted by using the tools of the Immune Epitope Database [14]. Spots were visualized with 3-amino-9-ethylcarbazole (BD Biosciences) and counted on an SZ61 stereomicroscope (Olympus, Tokyo, Japan).

## Intracellular cytokine staining

Unless otherwise noted, all antibodies and reagents were purchased from BioLegend. Single-cell suspensions from the spleen were stimulated for 2 h with or without 1 µg/mL PepMix SARS-CoV-2 S peptide pool 1+2 in DMSO [11] or 5 µg/mL SARS-CoV-2 S_268–276_ in DMSO/H_2_O (4:1, v/v), followed by the addition of 5 µg/mL brefeldin A for additional stimulation overnight. The cells were blocked with TruStain FcX anti-CD16/32 (clone no. 93, 5 µg/mL) and surface-stained with the following antibodies: anti-CD3ε phycoerythrin (PE)/Cy7 (clone no. 145-2C11, 0.25 µg/mL), anti-CD4 allophycocyanin (APC)/Fire 750 (clone no. GK1.5, 0.25 µg/mL), anti-CD8β peridinin chlorophyll protein (PerCP)/Cy5.5 (clone no. YTS156.7.7, 0.25 µg/mL), anti-CD62L fluorescein isothiocyanate (FITC) (clone no. MEL-14, 1.25 µg/mL), and anti-CD44 PE (clone no. IM7, 0.5 µg/mL). Intracellular staining was performed using the eBioscience intracellular fixation and permeabilization buffer set (Thermo Fisher Scientific) and the following antibodies: anti-IFN-γ APC (clone no. XMG1.2, 0.5 µg/mL), anti-IL-2 Brilliant Violet 510 (clone no. JES6-5H4, 1 µg/mL), and anti-TNF-α Brilliant Violet 421 (clone no. MP6-XT22, 0.5 µg/mL). A flow cytometric analysis was performed on a FACSVerse cytometer (BD Biosciences) with FlowJo software (BD Biosciences).

## Statistical analysis

Statistical analyses were performed using R software (R Foundation for Statistical Computing, Vienna, Austria). A *P*-value of less than 0.05 was considered to be significant.

References

1. Shahnaij M, Iyori M, Mizukami H, et al. Liver-Directed AAV8 Booster Vaccine Expressing Plasmodium falciparum Antigen Following Adenovirus Vaccine Priming Elicits Sterile Protection in a Murine Model. Front Immunol. 2021;12:612910.

2. Schneider CA, Rasband WS, Eliceiri KW. NIH Image to ImageJ: 25 years of image analysis. Nat Methods. 2012 Jul;9(7):671-5.

3. Wan J, Xing S, Ding L, et al. Human-IgG-Neutralizing Monoclonal Antibodies Block the SARS-CoV-2 Infection. Cell Rep. 2020 Jul 21;32(3):107918.

4. Nie J, Li Q, Wu J, et al. Establishment and validation of a pseudovirus neutralization assay for SARS-CoV-2. Emerg Microbes Infect. 2020 Dec;9(1):680-686.

5. Nie J, Li Q, Wu J, et al. Quantification of SARS-CoV-2 neutralizing antibody by a pseudotyped virus-based assay. Nat Protoc. 2020 Nov;15(11):3699-3715.

6. Shang J, Ye G, Shi K, et al. Structural basis of receptor recognition by SARS-CoV-2. Nature. 2020 May;581(7807):221-224.

7. Planas D, Veyer D, Baidaliuk A, et al. Reduced sensitivity of SARS-CoV-2 variant Delta to antibody neutralization. Nature. 2021 Aug;596(7871):276-280.

8. Mlcochova P, Kemp SA, Dhar MS, et al. SARS-CoV-2 B.1.617.2 Delta variant replication and immune evasion. Nature. 2021 Nov;599(7883):114-119.

9. Reynolds CJ, Pade C, Gibbons JM, et al. Prior SARS-CoV-2 infection rescues B and T cell responses to variants after first vaccine dose. Science. 2021 Apr 30.

10. Tscharke DC, Woo WP, Sakala IG, et al. Poxvirus CD8+ T-cell determinants and cross-reactivity in BALB/c mice. J Virol. 2006 Jul;80(13):6318-23.

11. Braun J, Loyal L, Frentsch M, et al. SARS-CoV-2-reactive T cells in healthy donors and patients with COVID-19. Nature. 2020 Nov;587(7833):270-274.

12. Tscherne A, Schwarz JH, Rohde C, et al. Immunogenicity and efficacy of the COVID-19 candidate vector vaccine MVA-SARS-2-S in preclinical vaccination. Proc Natl Acad Sci U S A. 2021 Jul 13;118(28).

13. Zhuang Z, Lai X, Sun J, et al. Mapping and role of T cell response in SARS-CoV-2-infected mice. J Exp Med. 2021 Apr 5;218(4).

14. Vita R, Mahajan S, Overton JA, et al. The Immune Epitope Database (IEDB): 2018 update. Nucleic Acids Res. 2019 Jan 8;47(D1):D339-D343.

**
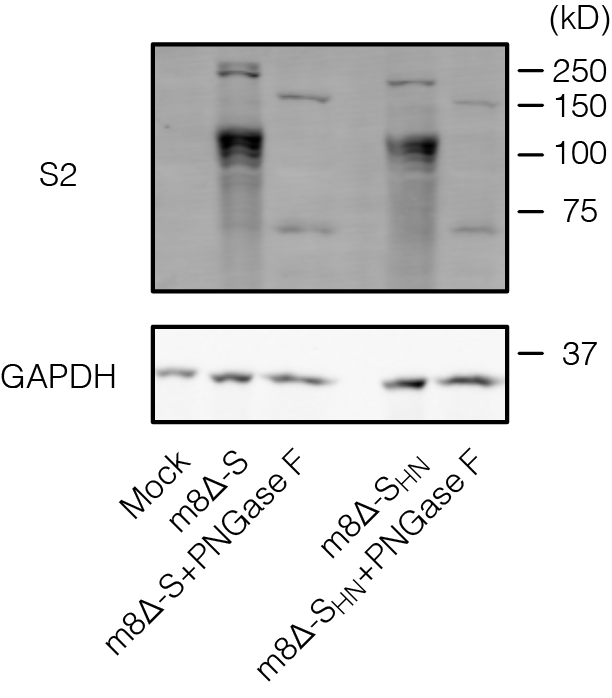
Supplementary Figure 1.** Glycosylation of the S protein, as determined by immunoblotting. HEK293T cells were inoculated with the indicated recombinant virus or were mock-infected one day before the analysis. N-glycans were removed using peptide-N_4_-(N-acetyl-β-D-glucosaminyl)asparagine amidase F (PNGase F). Antibody targets are shown on the left side of the panels. GAPDH was used as a loading control. m8Δ-S, m8Δ-SARS2(P7.5-S)-HA; m8Δ-S_HN_, m8Δ-SARS2(P7.5-S_HN_)-HA.

**
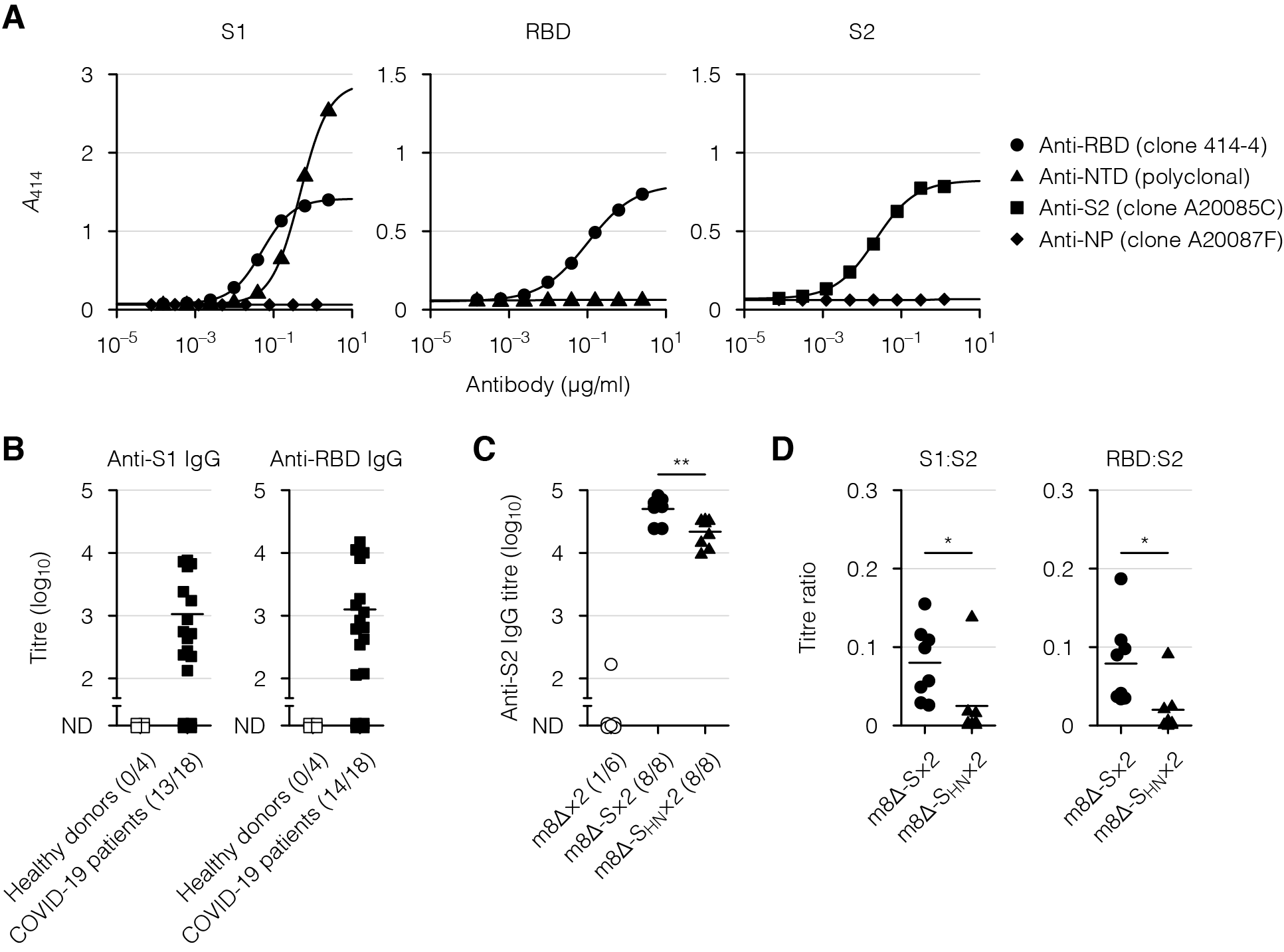
Supplementary Figure 2.** Supplementary ELISA data. (A) Validation of the recombinant S1, S2, and RBD proteins for ELISA. The indicated recombinant proteins were coated on ELISA plates, followed by incubation with serially diluted specific or irrelevant antibodies. *A*_414_, absorbance at 414 nm; NTD, N-terminal domain in the S1 domain; NP, nucleocapsid protein. (B) Endpoint titres of the indicated antibodies in serum samples obtained from healthy donors and COVID-19 patients. Titres of <100 are listed as not determined (ND). The proportions of samples with measurable titres are shown in parentheses. Each symbol represents an individual donor or patient. Horizontal lines represent geometric means. (C) Endpoint titres of the S2-specific IgG at six weeks after boost immunization with the indicated virus. The proportions of samples with measurable titres are shown in parentheses. Each symbol represents an individual mouse. Horizontal lines represent the geometric means. Data were pooled from three independent experiments using six to eight mice per experimental group. m8Δ-S, m8Δ-SARS2(P7.5-S)-HA; m8Δ-S_HN_, m8Δ-SARS2(P7.5-S_HN_)-HA. (D) Ratios of the S1-specific or RBD-specific IgG titre to S2-specific IgG titre in (C) and Figure 3(C). Each symbol represents an individual mouse. Horizontal lines represent the geometric means. The log-transformed data shown in (C) and the data shown in (D) were analysed by a Welch *t*-test. ***P* < 0.01, **P* < 0.05.


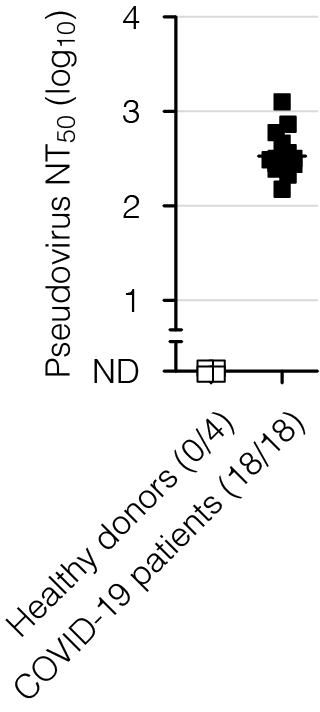
**Supplementary Figure 3.** Validation of the pseudovirus using serum samples from healthy donors and COVID-19 patients. Neutralizing activities of these serum samples against the Wuhan-Hu-1 pseudovirus were measured. The proportions of samples with measurable neutralizing activity are shown in parentheses. NT_50_, 50% neutralizing concentration; ND, not determined.

**
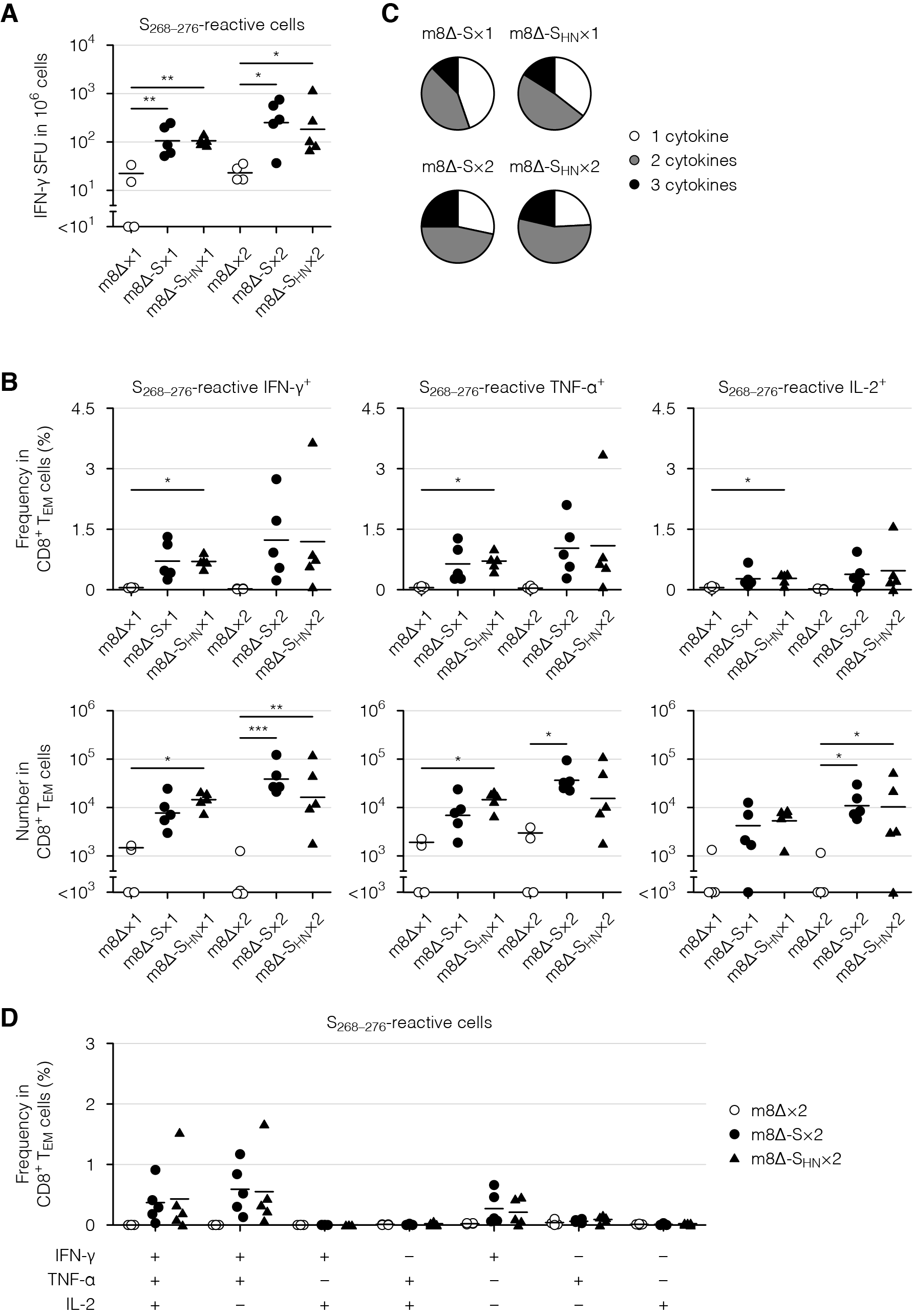
Supplementary Figure 4.** Cytokine profiles of the S_268–276_ peptide-reactive cells. (A) ELISpot assay of the S_268–276_ peptide-reactive cells. Splenocytes were obtained six weeks after the primary (×1) or second (×2) immunization with the indicated virus. IFN-γ spot-forming units (SFU) were measured after *in vitro* stimulation with the S_268–276_ peptide. Each symbol represents an individual mouse. Horizontal lines represent the geometric means. Data were pooled from two independent experiments using four to five mice per experimental group. m8Δ-S, m8Δ-SARS2(P7.5-S)-HA; m8Δ-S_HN_, m8Δ-SARS2(P7.5-S_HN_)-HA. (B) Frequencies and absolute numbers of the CD8^+^ effector memory T (T_EM_) cells reactive to the S_268–276_ peptide described in (A), as measured by intracellular cytokine staining. Each symbol represents an individual mouse. Horizontal lines represent the means (upper panels) or geometric means (lower panels). (C, D) Frequencies of the S_268–276_ peptide-reactive CD8^+^ T_EM_ cells described in (B) expressing the indicated number (C) or combination (D) of cytokines among IFN-γ, TNF-α, and IL-2. The log-transformed data in (A and B, lower panels) and data in (B, upper panels) after the primary and second immunizations were analysed individually by a Tukey test. Combinations without mark are not statistically different. ****P* < 0.001, ***P* < 0.01, **P* < 0.05.

**
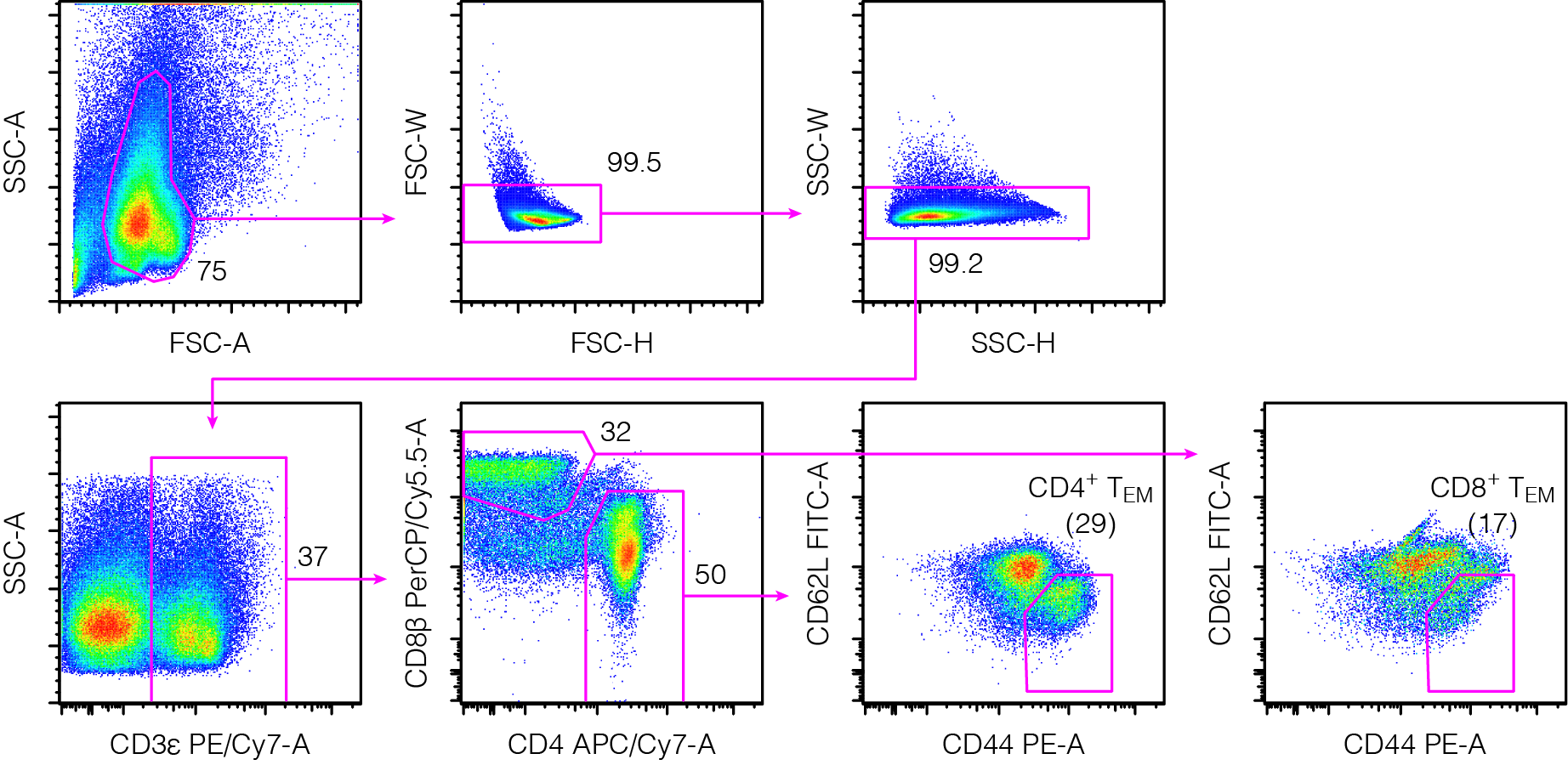
Supplementary Figure 5.** Gating strategy used to isolate the CD8^+^ and CD4^+^ effector memory T (T_EM_) cells. Shown are representative plots of splenocytes collected six weeks after boost immunization with m8Δ-SARS2(P7.5-S)-HA, following their *in vitro* stimulation with the S peptide mix. Numbers indicate the percentages of cells in each gate.

**
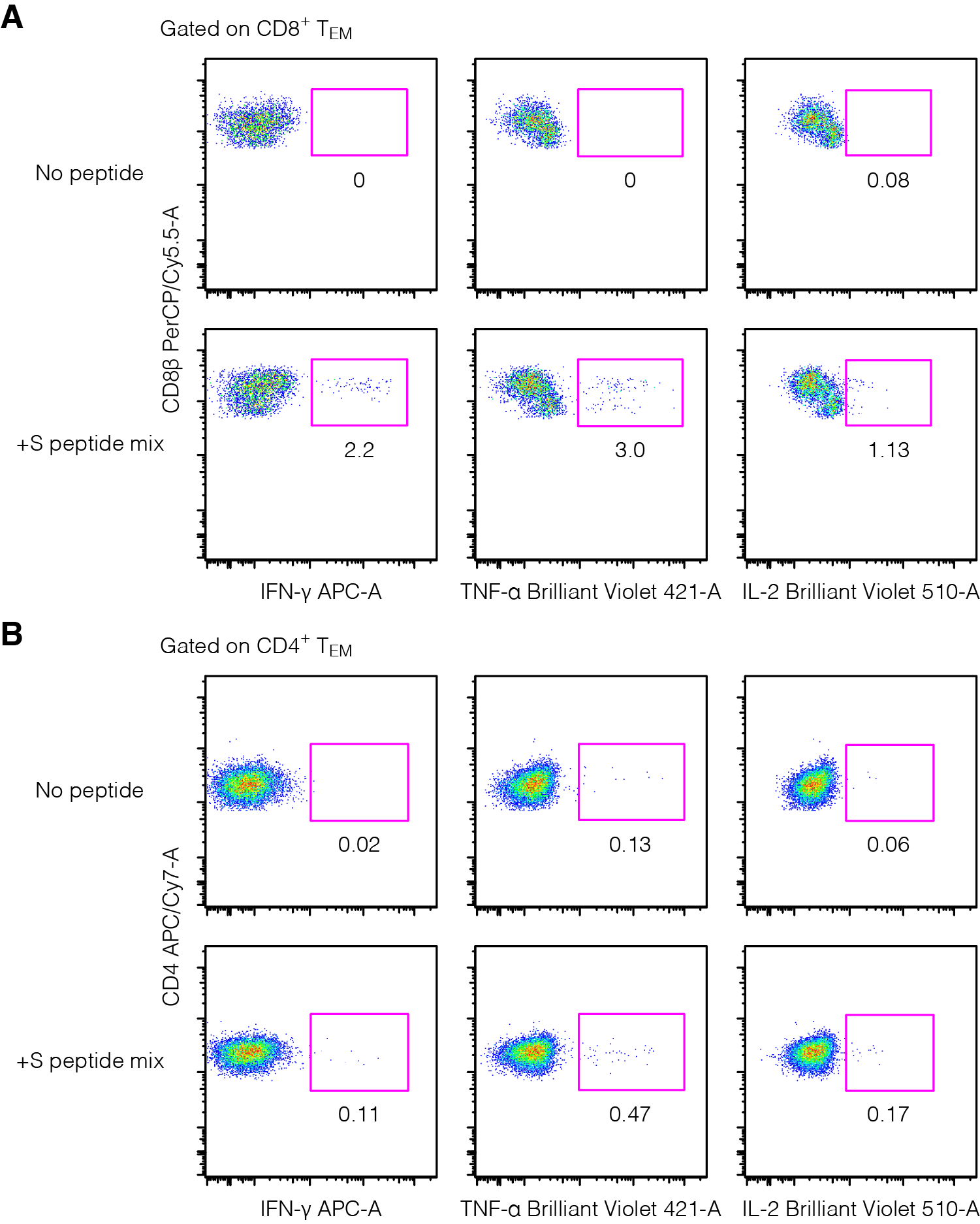
Supplementary Figure 6.** Gating strategy used to identify the cytokine-producing cells. Shown are representative plots of CD8^+^ (A) and CD4^+^ (B) effector memory T (T_EM_) cells collected from the spleen six weeks after boost immunization with m8Δ-SARS2(P7.5-S)-HA, following their *in vitro* stimulation or not with the S peptide mix. Numbers indicate the percentages of cells in each gate.


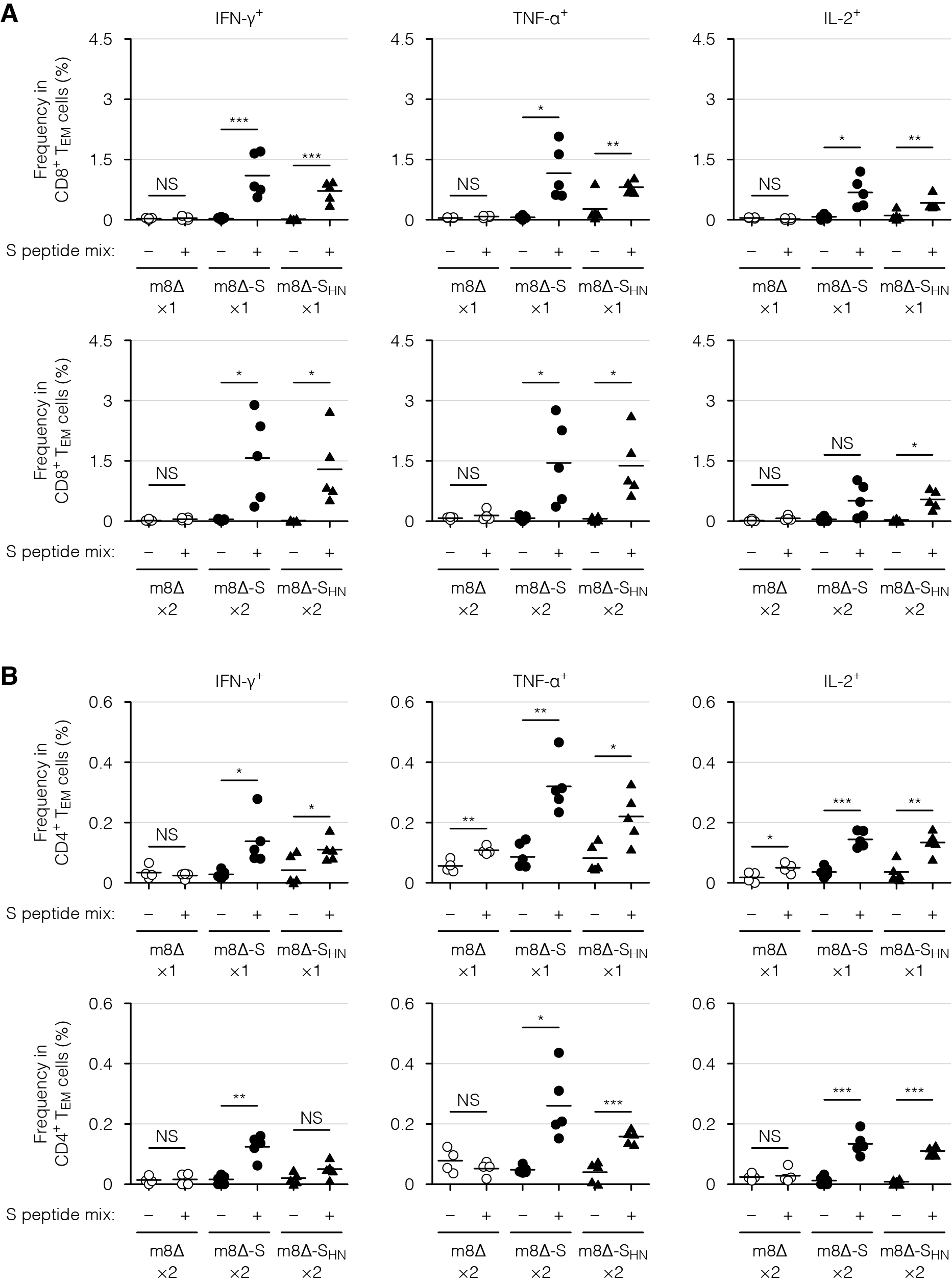
**Supplementary Figure 7**. Cytokine production by memory T cells. Frequencies of the cells producing the indicated cytokines were measured by the intracellular cytokine staining of the CD8^+^ (A) and CD4^+^ (B) effector memory T (T_EM_) cells stimulated (+) or not (–) with the S peptide mix. Splenocytes were obtained six weeks after the primary (×1) or boost (×2) immunization with the indicated virus. Each symbol represents an individual mouse. Horizontal lines represent the means. Data were pooled from two independent experiments using four or more mice per experimental group. m8Δ-S, m8Δ-SARS2(P7.5-S)-HA; m8Δ-S_HN_, m8Δ-SARS2(P7.5-S_HN_)-HA. The data were analysed by a Welch *t*-test. ****P* < 0.001, ***P* < 0.01, **P* < 0.05. NS, not statistically significant.

**
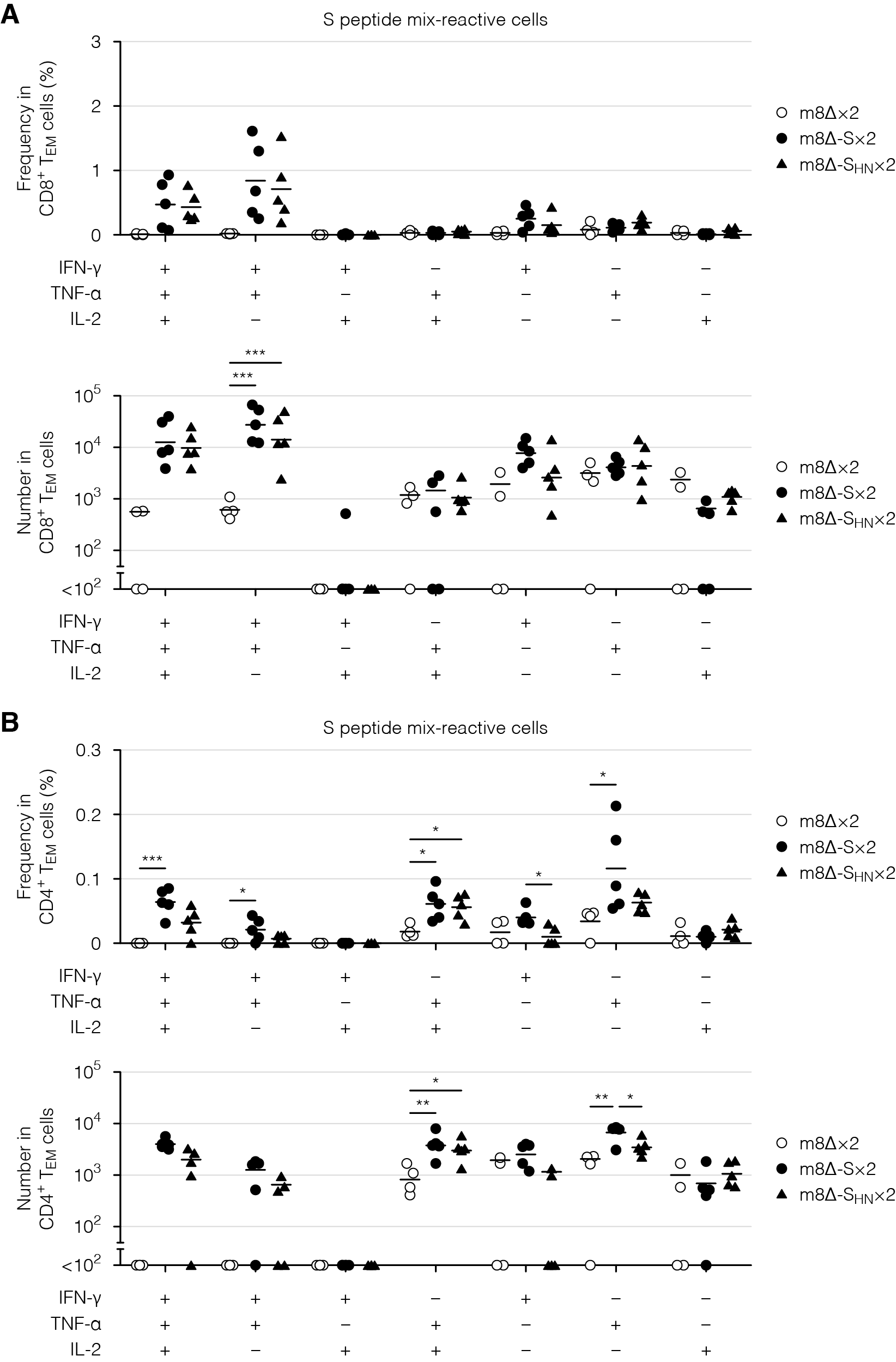
Supplementary Figure 8.** Detailed cytokine profiles of the effector memory T (T_EM_) cells, as measured by intracellular cytokine staining. Splenocytes were obtained six weeks after boost immunization with the indicated virus and stimulated *in vitro* with the S peptide mix. Shown are the frequencies and absolute numbers of the CD8^+^ (A) and CD4^+^ (B) T_EM_ cells expressing the indicated combination of cytokines. Each symbol represents an individual mouse. Horizontal lines represent the means (upper panels) or geometric means (lower panels). Data were pooled from two independent experiments using four to five mice per experimental group. m8Δ-S, m8Δ-SARS2(P7.5-S)-HA; m8Δ-S_HN_, m8Δ-SARS2(P7.5-S_HN_)-HA. The data in the upper panels and log-transformed data in the lower panels were analysed by a Tukey test among the individual combinations of cytokines. Combinations without mark are not statistically different. ****P* < 0.001, ***P* < 0.01, **P* < 0.05.
